# Multifractal and lacunarity features of retinal microvasculature in migraine: an optical coherence tomography angiography study

**DOI:** 10.1186/s12886-025-04407-y

**Published:** 2025-10-14

**Authors:** Abdülcemal Gürpınar, Yüksel Süllü

**Affiliations:** 1 Department of Ophthalmology, Ordu State Hospital, Ordu, 55200 Turkey; 2 Department of Ophthalmology, Ondokuz Mayıs University, Samsun, 55139 Turkey

**Keywords:** FD-300, Migraine, OCTA, Vascular tortuosity, Vascular diameter index, Branch point density, Fractal dimension, Macular lacunarity

## Abstract

**Purpose:**

To determine the multifractal and lacunarity characteristics of the retinal microvasculature in patients with migraine and compare with healthy controls.

**Methods:**

A total of 112 eyes from 56 migraine patients (35 MWO, 21 MWA) and 102 eyes from 51 healthy controls were included in the study. Optical coherence tomography angiography (OCTA) was used to assess foveal and parafoveal vascular parameters. Vascular area density, vascular length density, vascular diameter index, vascular tortuosity, branch point density, non-flow area, and foveal avascular zone parameters were measured with ImageJ. Fractal dimensions (D₀, D₁, D₂), multifractal spectrum (D(q)), and lacunarity (parameter *b*) were calculated using MATLAB.

**Results:**

Migraine with aura patients showed significantly reduced vascular area density, vascular length density, vascular diameter index, and branch point density values, particularly in the deep capillary plexus. The foveal avascular zone area and perimeter were significantly enlarged in the migraine with aura group. Fractal analysis revealed a significant decrease in D₀, D₁, and D₂ values in migraine with aura, especially in the deep capillary plexus. The multifractal spectrum (D(q)) exhibited a consistent downward shift in migraine with aura, suggesting global architectural simplification. Although not statistically significant, lacunarity analysis showed a trend toward increased spatial heterogeneity in migraine with aura, reflected by lower *b* values.

**Conclusion:**

Migraine, particularly with aura, is associated with reduced vascular complexity and increased spatial irregularity in the retinal microvasculature, especially at the level of the deep capillary plexus. Multifractal and lacunarity metrics may serve as sensitive indicators of subclinical microvascular disruption in migraine.

## Introduction

Migraine is a prevalent neurovascular disorder that affects nearly one billion individuals globally and is defined by recurrent headache attacks frequently associated with photophobia, phonophobia, and sensitivity to motion [1, 2]. Episodic migraine is classified into two main subtypes: migraine with aura (MA) and migraine without aura (MO). Visual aura, the most frequently encountered form, typically manifests as transient visual disturbances such as decreased visual acuity, photopsia, and scotomas [3]. 

The global prevalence of migraine is estimated at approximately 15%, with notably higher incidence rates observed among women, especially at younger ages [4]. Its prevalence is highest among Caucasians, with a peak occurrence in individuals aged 18–44 years. Migraine represents a significant cause of disability, profoundly impacting social functioning, workforce productivity, mental health, academic performance, and career advancement during the most productive years of life [5]. 

Embryologically, the retina and optic nerve develop as extensions of the diencephalon, thus closely resembling the central nervous system regarding anatomical structure, functional characteristics, injury response, and immunological properties [6]. Additionally, the retina and cerebral cortex share analogous mechanisms in angiogenesis and blood flow regulation [7]. Consequently, it is reasonable to describe the eye as an extension of the brain.

The retinal microvascular structure can be characterized by its complex geometry and inherent fractal properties. In this context, fractal dimension and lacunarity analyses are effective quantitative methods for assessing the retinal vascular network’s structural complexity and spatial homogeneity [8]. Fractal dimension quantifies the intricacy of the retinal microvasculature, whereas lacunarity evaluates tissue heterogeneity by analyzing the distribution and irregularity of gaps within the vascular pattern [9]. Recent studies have demonstrated that these analytical approaches may serve as potential biomarkers for the diagnosis and monitoring of various systemic conditions and neurodegenerative disorders, including diabetic and hypertensive retinopathy [10–12]. Thus, evaluating the fractal and lacunarity characteristics of the retinal microvasculature offers novel insights into both ophthalmologic and systemic disease mechanisms.

Previous optical coherence tomography angiography (OCTA) studies in migraine have primarily examined alterations in foveal and parafoveal vessel density and foveal avascular zone (FAZ) dimensions. Most have reported reductions in these parameters, especially in patients with aura [13, 14]. In the present study, we conducted a comprehensive evaluation of all microvascular indices and overall vascular architecture obtained from 6 × 6 mm macular angiograms in migraine patients and compared them with those of healthy controls to identify potential differences.

## Materials and methods

### Study design and ethical approval

Between November 2023 and September 2024, patients over 18 who presented to the Ophthalmology Clinic of Ondokuz Mayıs University and were diagnosed with migraine were prospectively enrolled in the study. Age- and sex-matched healthy volunteers were included as the control group (CG). Informed consent was obtained from all participants, and OCTA imaging was performed voluntarily. Ethical approval for the study was obtained from the Clinical Research Ethics Committee of Ondokuz Mayıs University (approval no: 202400368), and the study adhered to the tenets of the Declaration of Helsinki.

### Study participants

Patients over 18 years of age with a confirmed diagnosis of migraine by a neurologist according to the International Classification of Headache Disorders, 3rd edition (ICHD-3) were prospectively included. Patients were excluded if they had a history of ocular trauma or congenital/acquired ocular pathology, systemic diseases that could affect the retinal vasculature (e.g., hypertension, diabetes, hyperthyroidism), a diagnosis of migraine secondary to intracranial pathology, OCTA images with an image quality value below 60, or were over 65 years of age. Age- and sex-matched healthy individuals were recruited from volunteers who had no history of migraine or other primary headache disorders, confirmed by neurological evaluation. For all participants, demographic data (age, sex), systemic and ocular comorbidities, duration of migraine history, and attack frequency were documented. In addition, use of vasoactive medications (e.g., beta-blockers, antiepileptics, calcium channel blockers) was recorded, and patients receiving such treatments were excluded.

### Clinical data and imaging

Participants were categorized into MWO and MWA groups based on clinical diagnosis. The diagnosis and subgroup classification were confirmed by a neurologist. Information regarding migraine duration, presence of aura, and associated symptoms was also obtained through a detailed history by the same neurologist. All OCTA scans were acquired during the interictal period, defined as at least 24 h after the last migraine attack and at least 24 h before the next expected attack.

For all participants, age, sex, systemic and ocular comorbidities, visual acuity, intraocular pressure (IOP), central corneal thickness (CCT), axial length (AL), and spherical equivalent (SE) were recorded for each eye. Best-corrected visual acuity (BCVA) was measured using a Snellen chart and converted to logMAR for statistical analysis. IOP was measured by Goldmann applanation tonometry. CCT and AL measurements were obtained using the Zeiss IOL Master 500 (Carl Zeiss Meditec, Jena, Germany). A comprehensive anterior segment and fundus examination was conducted via slit-lamp biomicroscopy to exclude ocular pathologies.

Macular OCT, retinal nerve fiber layer (RNFL) thickness, and 6 × 6 mm macular OCTA images were acquired using the Topcon DRI SS-OCT Triton (Topcon, Tokyo, Japan). The device software automatically segments retinal layers and allows for choroidal thickness measurement. Using the OCTARA algorithm, the software automatically segments the superficial capillary plexus (SCP), deep capillary plexus (DCP), outer retina, and choriocapillaris. The SCP is segmented from the internal limiting membrane (ILM) to a depth of 15.6 μm into the inner plexiform/nuclear layers, while the DCP is segmented between 15.6 μm and 70.2 μm within the same layers. Image quality is assessed automatically by the system, and images with an image quality value (IQV) of at least 60 were included in the analysis.

### Magnification correction

Magnification effects caused by the eye and the imaging system can lead to an overestimation of the dimensions of an OCTA angiogram. A correction method based on AL was applied to ensure accurate calculation of ocular parameters using the Littmann and Bennett formulas. The Littmann formula is expressed as $\:D\text{t}\:=\:p\:\times\:\:q\:\times\:\:D\text{m}$, where $\:D\text{t}$ is the true dimension, $\:D\text{m}\:$ is the measured dimension, $\:p$ represents the magnification from the imaging system, and $\:q$ accounts for ocular magnification [15]. Ocular magnification (q) is calculated using the Bennett formula: $\:q\:=\:0.01306\:\times\:(AL-1.82)$, where 1.82 is a constant related to the distance from the corneal apex to the second principal plane of the eye [15]. In our imaging system, the AL at which the actual and measured dimensions are equal ($\:D\text{t}\:=\:D\text{m}$) is 24.39 mm, which corresponds to a magnification factor of $\:p=1/q$. Accordingly, p is calculated as $\:p=1/0.01306\:\times\:\:(24.39\:-\:1.82)$ $\:=3.39$. Based on this, the magnification factor for each angiogram area was determined using the formula $\:D\text{t}^{2}/D\text{m}^{2}\:=\:\text{0,001960}\:\times\:\:(AL-\text{1,82})^{2}$[16]. 

### OCT and OCTA data processing

For each participant, vertical cup-to-disc ratio, ganglion cell complex (GCC) thickness in nine quadrants, RNFL thickness, disc area, and rim area were recorded. SCP and DCP vessel densities in the foveal and parafoveal regions were measured using the ETDRS grid. All values were obtained from the automated measurements of the Topcon DRI SS-OCT Triton software.

From 6 × 6 mm SCP and DCP en-face angiograms, the following vascular parameters were calculated using ImageJ [17]: vessel area density (VAD), vessel length density (VLD), vessel diameter index (VDI), vessel tortuosity (VT), branch point density (BD), non-flow area (NFA), FAZ area, FAZ perimeter, FAZ irregularity index, FAZ axis ratio, and FD-300 (vessel density within 300 μm of the FAZ boundary). Fractal dimension (FD) and lacunarity values for macular SCP, DCP, and peripapillary angiograms were calculated using MATLAB R2024a (The MathWorks Inc., Natick, MA, USA). Vascular and FAZ parameters were defined as recommended by Sampson et al.^18^

### ImageJ-Based OCTA image analysis

**Fig. 1:**
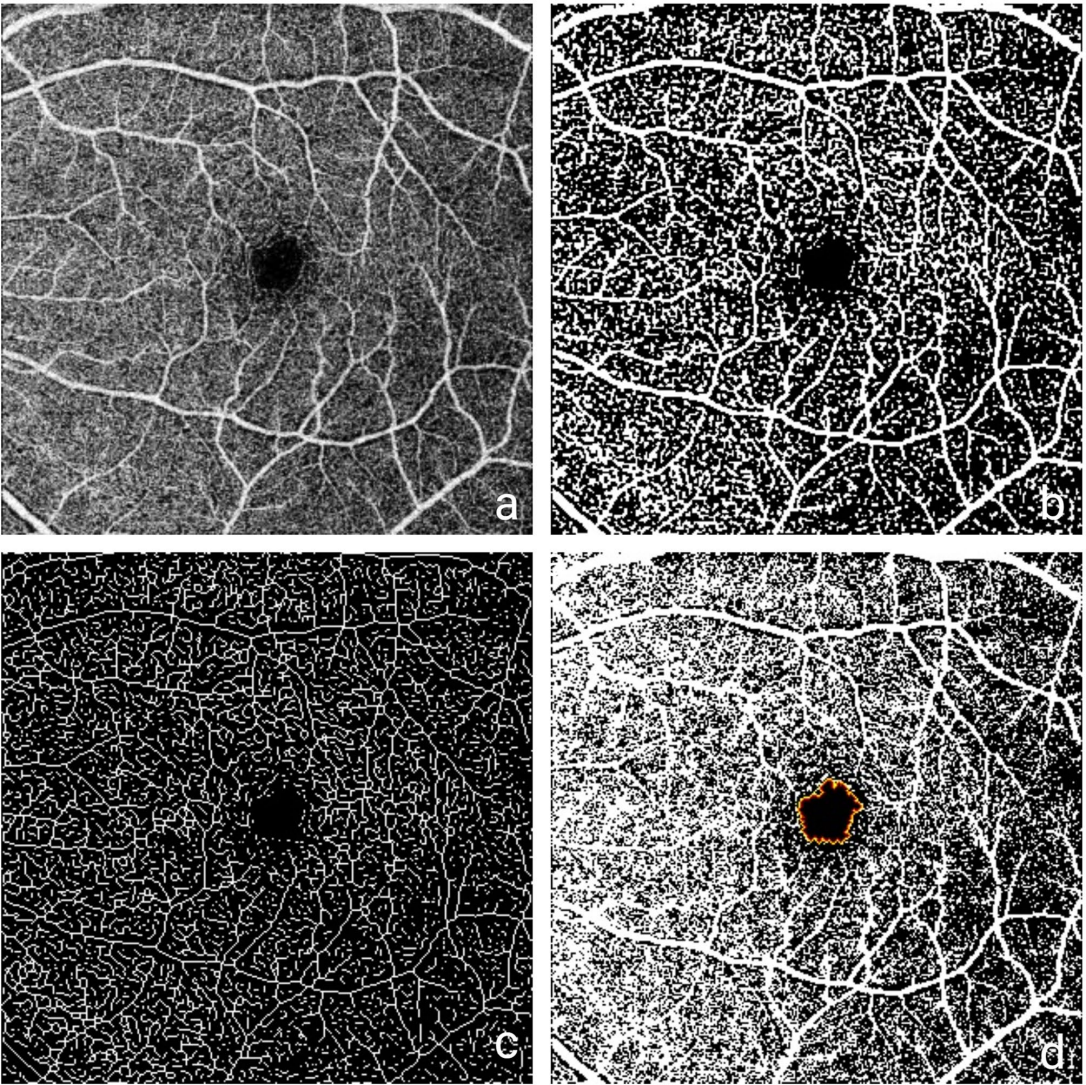
Processing steps for retinal OCTA images using ImageJ: (a) magnification-corrected 6 × 6 mm angiogram, (b) binarized image, (c) skeletonized image, (d) FAZ segmentation

Macular angiograms measuring 6 × 6 mm were initially corrected using axial length–adjusted magnification factors and then converted to an 8-bit format. Motion artifacts were first corrected using the “TurboReg” plugin in ImageJ to calculate VAD and NFA. Then, background equalization was performed using the “Subtract Background” function. A Gaussian filter was then applied to the images, and binarization was performed using the Otsu thresholding method [18, 19]. To assess the FAZ area, FAZ perimeter, FAZ irregularity index, and axis ratio (the ratio of the major to minor axis of the fitted ellipse), as well as the FD-300 values, the angiograms were binarized using the aforementioned preprocessing steps. Then, the “Level Sets” plugin was used to automatically delineate the FAZ contour by identifying the black pixel region surrounded by white pixels on the binary image (Fig. 1) [20]. The FAZ irregularity index (also referred to as circularity deviation) was computed as the ratio of the measured FAZ perimeter to the theoretical perimeter of a perfect circle with the same area, using the formula: $\:FAZ\:perimeter/2\sqrt{\pi\:\:\times\:\:FAZ\:area}$. To measure VLD, VDI, BD, and VT, binarized images were skeletonized using the “Skeletonize” function, converting the vessels into one-pixel-wide representations (Fig. 1).

### Fractal dimension and lacunarity analysis

**Fig. 2:**
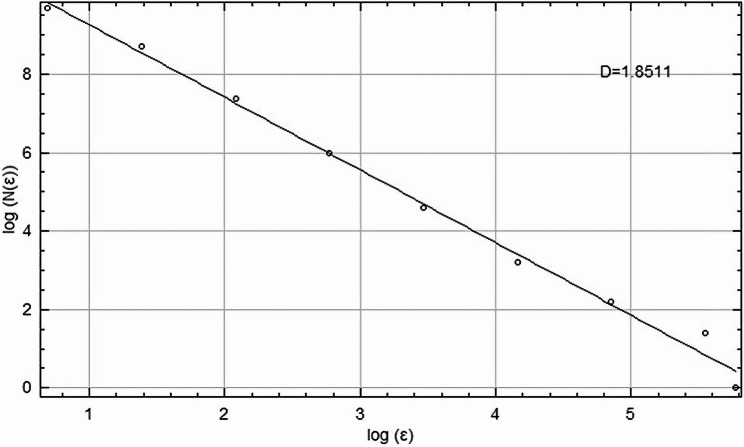
Fractal analysis using box-counting method: log(N(ε)) vs. log(ε) plot

The FD was calculated using the classical box-counting method. The binarized angiogram was overlaid with grids of boxes of decreasing size (ε), and the number of boxes containing vessel segments (N(ε)) was counted. FD (D₀) was defined as the slope of the linear region in the log–log plot of log(N(ε)) versus log(ε) (Fig. 2) [21]. The box sizes used were 2, 4, 8, 16, 32, and 64 pixels. Calculations were performed using MATLAB R2024a.

Lacunarity was analyzed using a gliding box algorithm in MATLAB. The lacunarity function was modeled as a hyperbolic equation: L(ε) = b/ε^a^ + c.^22^ The “b” parameter was used as the lacunarity index, where lower b-values indicated higher lacunarity.

In addition to classical box-counting analysis, multifractal dimensions including D₀, D₁, D₂, D_−5_, and D₅ were calculated from SCP and DCP angiograms using the same MATLAB framework. From the resulting multifractal spectrum, additional spectral parameters were derived: Δf (width of the f(α) spectrum), Δα (range of Hölder exponents), and A (asymmetry index of the spectrum). The Δf value reflects the degree of multifractality; Δα represents the range of singularities, and A describes the skewness of the spectrum, with A > 1 indicating left-skewed and A < 1 indicating right-skewed distributions. These parameters were computed separately for SCP and DCP layers using q-values ranging from − 5 to + 5 in 0.5 increments [22]. 

### Statistical analysis

Statistical analyses were performed using SPSS version 26.0 (IBM Corp., Armonk, NY, USA). The normality of the distribution of continuous variables was assessed using the Shapiro-Wilk and Kolmogorov-Smirnov tests. Normally distributed variables were presented as mean ± standard deviation, whereas non-normally distributed variables were expressed as median (minimum–maximum).To compare the three groups, one-way ANOVA was used for normally distributed variables, and the Kruskal-Wallis test was applied for non-normally distributed variables. A p-value of < 0.05 was considered statistically significant. Categorical variables were analyzed using the chi-square test. In cases where a significant overall difference among groups was found, post hoc analysis was conducted to identify the source of the difference. Tukey’s HSD test was used for normally distributed variables when variances were homogeneous, and the Dunnett T3 test was applied when variances were not homogeneous. For non-normally distributed variables, pairwise comparisons were performed using the Bonferroni-corrected Mann–Whitney U test, with a significance level set at *p* ≤ 0.0167.

## Results

A total of 112 eyes from 56 migraine patients (35 MWO, 21 MWA) and 102 eyes from 51 healthy controls were included in the study. Demographic and baseline ocular characteristics of the participants are presented in Table 1. There were no significant differences among the three groups regarding age, gender, BCVA, IOP, CCT, SE, or AL (all *p* > 0.05).

**Table 1:** Demographic and clinical characteristics of participants

Variables	MWO (*n* = 35)	MWA (*n* = 21)	Control (*n* = 51)	*p*-value
	Mean ± SD or Median (min-max)			
Age	33 (18–59)	32.48 ± 10.56	29 (20–59)	0.848^a^
Gender n. %				
Female	23 (65.7)	14 (66.7)	34 (66.7)	0.995^c^
Male	12 (34.3)	7 (33.3)	17 (33.3)	
Duration of migraine history (months)	48 (6–180)	48 (8–140)	–	0.158^b^
Migraine days/month	5 (2–9)	6 (2–14)		0.456^b^
BCVA (logMAR, *n* = 214 eye)	0.00 (0.00–0.10)	0.00 (0.00–0.06)	0.00 (0.00–0.06)	0.173^a^
IOP (*n* = 214 eye)	15.48 ± 1.82	14.53 ± 2.39	14.85 ± 1.73	0.075^d^
CCT (*n* = 214 eye)	545.42 ± 27.27	545.33 ± 32.48	520 (480–587)	0.087^a^
C/D (*n* = 214 eye)	0.52 (0.10–0.79)	0.60 (0.14–0.74)	0.47 ± 0.16	0.120^a^
SE (Diopters, *n* = 214 eye)	−0.15 ± 1.28	−0.30 ± 0.87	−0.50 (−1.50–1.25)	0.245^a^
AL (mm, *n* = 214 eye)	23.19 ± 0.68	23.50 (21.59–24.32)	23.37 ± 0.75	0.348^a^

*MWO* migraine without aura, *MWA* migraine with aura, *BCVA* best corrected visual acuity, *IOP* intraocular pressure, *CCT* central corneal thickness, *C/D* cup to disc ratio, *SE* spherical equivalent, *AL* axial length, a: Kruskal-Wallis test, b: Mann Whitney U test, c: Chi-square test, d: One Way ANOVA test

### Macular vascular density

The SCP foveal vascular density was significantly lower in the MWA group than in the MWO and control groups (p_1_ = 0.001; p_2_ = 0.001), while no significant difference was found between the MWO and control groups (p_3_ = 0.274). Among the parafoveal quadrants of the SCP, only the inferior quadrant showed a significant difference across groups (*p* = 0.010), where post-hoc analysis revealed a significantly lower VD in the MWA group compared to controls (p_2_ = 0.003). Table 2 shows the VD values of the SCP and DCP layers in the foveal and parafoveal regions.

**Table 2:** Comparative analysis of vascular density parameters

Variables	MWO (*n* = 70 eyes)	MWA (*n* = 42 eyes)	Control (*n* = 102 eyes)	*p*-value
	Mean ± SD or Median (min-max)			
SCP Foveal VD (%)	20.06 ± 4.31	16.85 ± 3.70	21.02 ± 3.88	**0.001** ^**b**^ **p**_**1**_ **= 0.001** **p**_**2**_ **= 0.001** p_3_ = 0.274
SCP Parafoveal VD (%)				
Superior	47.45 (37.38–50.93)	46.19 ± 3.38	47.35 ± 2.63	0.231^a^
Nasal	47.12 (36.04–50.79)	46.25 ± 2.74	47.03 ± 2.21	0.351^a^
Inferior	47.39 (36.69–54.48)	46.24 (36.45–51.62)	47.39 ± 2.76	**0.010** ^**a**^ p_1_ = 0.034 **p**_**2**_ **= 0.003** p_3_ = 0.239
Temporal	48.06 (38.59–51.71)	47.34 ± 2.01	47.81 ± 2.43	0.352^a^
DCP Foveal VD (%)	16.81 ± 4.00	14.95 ± 4.17	18.70 ± 3.60	**0.001** ^**b**^ **p**_**1**_ **= 0.048** **p**_**2**_ **= 0.001** **p**_**3**_ **= 0.008**
DCP Parafoveal VD (%)				
Superior	48.29 (34.48–52.41)	47.56 ± 3.02	49.12 ± 2.80	**0.010** ^**a**^ p_1_ = 0.323 **p**_**2**_ **= 0.007** p_3_ = 0.033
Nasal	47.92 ± 2.94	47.58 (42.52–56.01)	49.19 ± 2.53	**0.002** ^**a**^ p_1_ = 0.433 **p**_**2**_ **= 0.003** **p**_**3**_ **= 0.006**
Inferior	48.04 (40.97–52.46)	47.56 (42.69–52.88)	49.10 (38.80–55.71)	**0.001** ^**a**^ p_1_ = 0.296 **p**_**2**_ **= 0.001** **p**_**3**_ **= 0.001**
Temporal	47.64 ± 2.47	47.32 ± 3.04	48.55 ± 2.87	**0.023** ^**b**^ p_1_ = 0.833 **p**_**2**_ **= 0.045** p_3_ = 0.089

*MWO* migraine without aura, *MWA* migraine with aura, *SCP* superficial capillary plexus, *DCP* deep capillary plexus, *VD* vascular density, a: Kruskal-Wallis test, b: One Way ANOVA test, p1: the difference between MWO and MWA group, p_2_: the difference between MWA and control group, p3: the difference between MWO and control group, p_1,_p_2,_ and p_3_ represent the adjusted p-values.

### Quantitative vascular parameters

**Fig. 3:**
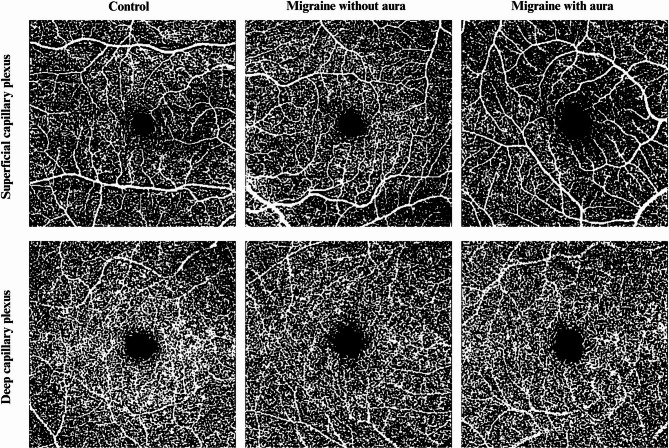
Representative binarized OCTA images of the macular microvasculature across study groups. (Top row: Superficial capillary plexus (SCP); Bottom row: Deep capillary plexus (DCP). Columns show a representative 6×6 mm binarized OCTA image from (left to right) a control subject, a migraine without aura (MWO) patient, and a migraine with aura (MWA) patient. In MWA, reduced capillary density and enlarged FAZ are notable, particularly in the DCP layer, suggesting microvascular rarefaction.)

Table 3 shows the quantitative vascular parameters derived from binarized and skeletonized en-face angiograms. In the SCP, the MWA group had significantly lower VAD, VLD, VDI, and BD than the controls (all p_2_ < 0.05), while NFA showed a trend without reaching statistical significance. In the DCP, VAD, VLD, and VDI were also significantly reduced in the MWA group compared to both MWO and control groups (all p_2_ and p_3_ < 0.05). NFA was significantly higher in the MWA group (*p* = 0.002). Figure 3 illustrates representative binarized OCTA images showing reduced capillary density and enlarged FAZ in MWA, especially within the deep capillary plexus.

**Table 3:** Comparative analysis of quantitative vascular parameters

Variables	MWO (*n* = 35)	MWA (*n* = 21)	Control (*n* = 51)	*p*-value
	Mean ± SD or Median (min-max)			
SCP				
VAD	34.41 (23.05–39.17)	32.91 ± 2.48	34.97 (25.45–41.26)	**0.006**^**a**^ p_1_ = 0.061 **p**_**2**_ **= 0.002** p_3_ = 0.129
VLD	8.96 (5.06–10.76)	8.50 ± 1.01	9.06 ± 1.18	**0.007**^**a**^ p_1_ = 0.205 **p**_**2**_ **= 0.004** p_3_ = 0.037
VDI	3.85 (3.55–4.56)	3.89 (3.54–4.47)	3.78 (3.28–4.47)	**0.005** ^**a**^ p_1_ = 0.678 **p**_**2**_ **= 0.009** **p**_**3**_ **= 0.007**
VT	1.172 ± 0.015	1.166 ± 0.014	1.176 (1.068–1.211)	0.055^a^
BD	2.43 (1.64–3.01)	2.39 ± 0.16	2.47 ± 0.17	**0.020** ^**a**^ p_1_ = 0.352 **p**_**2**_ **= 0.008** p_3_ = 0.071
NFA	23.20 (21.36–27.29)	23.74 ± 0.95	23.21 (21.15–25.23)	0.051^a^
DCP				
VAD	39.80 ± 2.07	39.15 ± 2.41	42.72 (34.32–45.46)	**0.001** ^**a**^ p_1_ = 0.115 **p**_**2**_ **= 0.001** **p**_**3**_ **= 0.001**
VLD	11.79 ± 0.97	11.56 ± 1.18	12.76 ± 1.04	**0.002** ^**b**^ p_1_ = 0.478 **p**_**2**_ **= 0.001** **p**_**3**_ **= 0.001**
VDI	3.35 (3.16–3.93)	3.34 ± 0.54	3.22 (2.97–3.65)	**0.001** ^**a**^ p_1_ = 0.043 **p**_**2**_ **= 0.001** **p**_**3**_ **= 0.001**
VT	1.243 ± 0.012	1.227 (1.217–1.30)	1.254 (1.20–1.28)	0.136^a^
BD	2.72 ± 0.11	2.73 ± 0.11	2.66 (2.01–3.28)	0.092^a^
NFA	20.91–0.89	21.37 ± 0.84	20.91 ± 0.55	**0.002** ^**b**^ **p**_**1**_ **= 0.005** **p**_**2**_ **= 0.002** p_3_ = 0.981

*MWO* migraine without aura, *MWA* migraine with aura, *SCP* superficial capillary plexus, *DCP* deep capillary plexus, *VAD* vascular area density, *VLD* vascular length density, *VT* vascular tortuosity, *BD *branch point density, *NFA* non-flow area, a: Kruskal Wallis test, b: One Way ANOVA test, p_1_:the difference between MWO and MWA group, p_2_: the difference between MWA and control group, p_3_ = the difference between MWO and control group, p_1,_p_2,_ and p_3_ represent the adjusted p-values.

### FAZ-Related parameters

The MWA group exhibited significantly higher FAZ area and perimeter values in the SCP compared to controls (*p* = 0.005 and *p* = 0.014, respectively). In the DCP, the MWA group had significantly higher FAZ area, perimeter, and circularity index values than the control group (p_2_ = 0.007, p_2_ = 0.006, and p_2_ = 0.001, respectively), while FD-300 was significantly lower (p_2_ = 0.012). Table 4 summarizes the FAZ metrics.

**Table 4:** Comparative analysis of FAZ-related and perifoveal vascular parameters

Variables	MWO (*n* = 35)	MWA (*n* = 21)	Control (*n* = 51)	*p*-value
	Mean ± SD or Median (min-max)			
SCP				
FAZ area (mm^2^)	0.44 ± 0.14	0.50 ± 0.19	0.39 ± 0.11	**0.001**^**d**^ p_1_ = 0.280 **p**_**2**_ **= 0.005** p_3_ = 0.058
FAZ perimeter (mm)	3.40 ± 0.70	3.58 ± 0.96	3.20 ± 0.63	**0.015**^**d**^ p_1_ = 0.420 **p**_**2**_ **= 0.014** p_3_ = 0.213
FAZ acircularity index	1.44 ± 0.13	1.42 (1.17–1.98)	1.40 ± 0.12	0.069^a^
FAZ axis ratio	1.17 ± 0.15	1.19 ± 0.16	1.16 ± 0.09	0.482^d^
FD-300 (%)	32.17 ± 3.54	30.94 ± 3.23	32.08 ± 4.13	0.197^d^
DCP				
FAZ area (mm^2^)	0.58 ± 0.18	0.63 ± 0.18	0.53 ± 0.15	**0.005**^**d**^ p_1_ = 0.520 **p**_**2**_ **= 0.007** p_3_ = 0.131
FAZ perimeter (mm)	4.36 ± 1.01	4.39 ± 0.80	3.90 ± 0.73	**0.001**^**d**^ p_1_ = 0.993 **p**_**2**_ **= 0.006** **p**_**3**_ **= 0.002**
FAZ acircularity index	1.55 (1.37–1.94)	1.58 ± 0.14	1.48 (1.27–1.96)	**0.001**^**a**^ p_1_ = 0.873 **p**_**2**_ **= 0.001** **p**_**3**_ **= 0.001**
FAZ axis ratio	1.17 ± 0.15	1.20 ± 0.14	1.13 (1.02–1.74)	0.169^a^
FD-300 (%)	34.19 ± 4.14	33.88 ± 4.99	36.34 ± 5.03	**0.003**^**d**^ p_1_ = 0.943 **p**_**2**_ **= 0.012** p_3_ = 0.017

*MWO* migraine without aura, *MWA* migraine with aura, *BCVA* best corrected visual acuity, *IOP* intraocular pressure, *CCT* central corneal thickness, *C/D* cup to disc raito, *SE* spherical equivalent, *AL* axial lenght, a: Kruskal Wallis test, b: Mann Whitney U test, c: Chi-square test, d: One Way ANOVA test, p_1_:the difference between MWO and MWA group, p_2_: the difference between MWA and control group, p_3_ = the difference between MWO and control group, p_1,_p_2_ and p_3_ represent the adjusted p-values.

### Fractal dimension and multifractal analysis

**Fig. 4:**
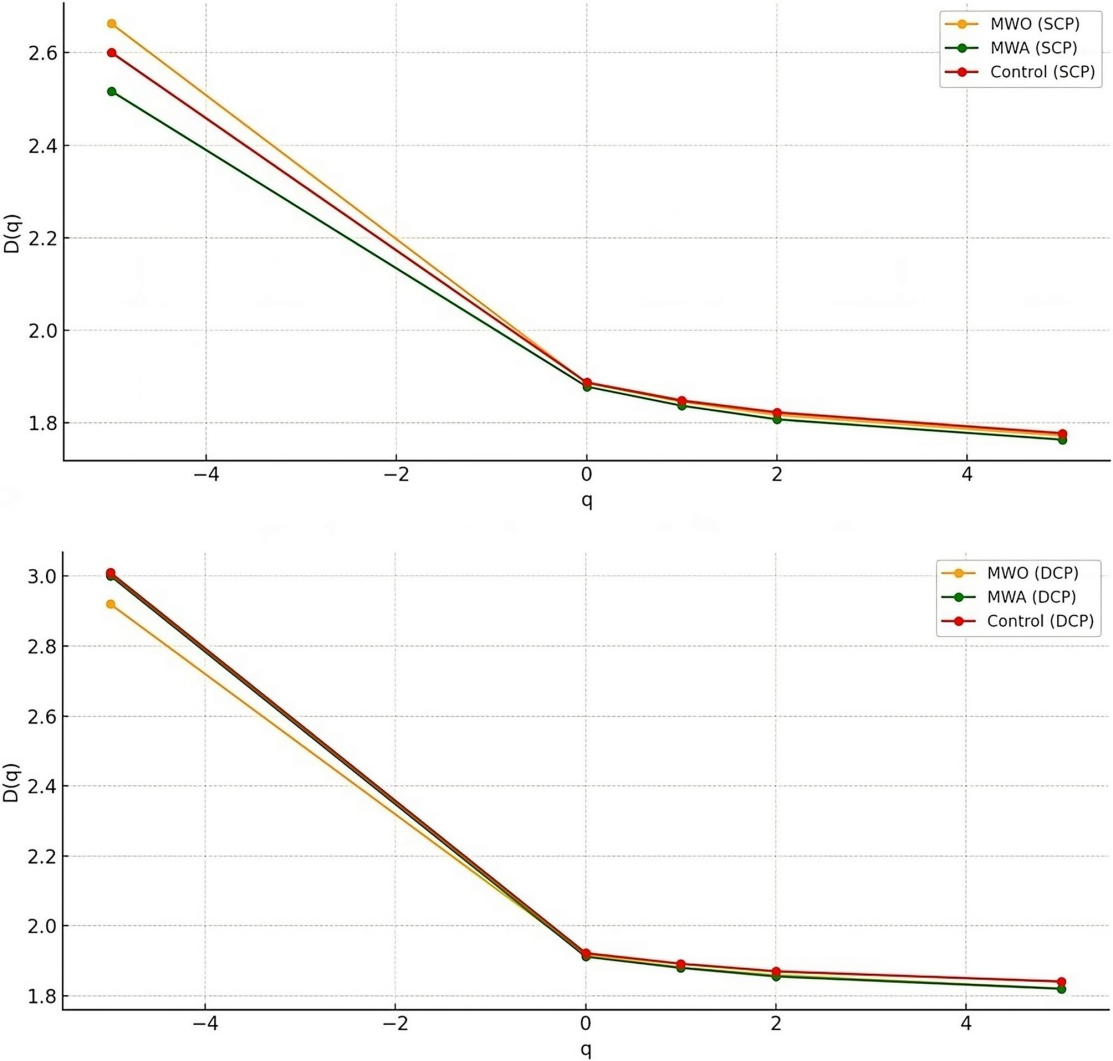
Comparison of D(q) curves in migraine and control groups

The MWA group exhibited significantly lower fractal dimensions (D0, D1, and D2) in both SCP and DCP compared to controls (p_2_ values < 0.01). D5 values were reduced in both migraine groups. However, other multifractal spectrum-derived parameters such as Δf, Δα, and asymmetry index (A) did not show significant group differences (Table 5). Figure 4 displays the multifractal D(q) spectra for SCP and DCP. A monotonic decrease in D(q) with increasing q-values was noted in all groups. The MWA group showed consistently lower D(q) values, particularly in the negative q range.

**Table 5:** Multifractal and lacunarity parameters in SCP and DCP layers across study groups

Variables	MWO (*n* = 35)	MWA (*n* = 21)	Control (*n* = 51)	*p*-value
	Mean ± SD or Median (min-max)			
Fractal dimension				
SCP D_0_	1.8864 (1.7186–1.9051)	1.8786 (1.7927–1.9043)	1.8875 (1.8095–1.9188)	**0.012** ^**a**^ p_1_ = 0.222 **p**_**2**_ **= 0.009** p_3_ = 0.624
SCP D_1_	1.8457 (1.7307–1.8698)	1.8373 (1.7626–1.8671)	1.8485 (1.7558–1.8871)	**0.007** ^**a**^ p_1_ = 0.284 **p**_**2**_ **= 0.006** p_3_ = 0.356
SCP D_2_	1.8173 (1.6943–1.8445)	1.8079 (1.7479–1.8397)	1.8228 (1.7191–1.8641)	**0.002** ^**a**^ p_1_ = 0.541 **p**_**2**_ **= 0.003** p_3_ = 0.075
SCP D_−5_	2.6630 (1.3569–3.9445)	2.5170 (2.0649–3.9551)	2.6006 (2.0594–3.9710)	0.998^a^
SCP D_5_	1.7725 (1.6472–1.8083)	1.7640 ± 0.0268	1.7777 ± 0.0286	**0.003** ^**a**^ p_1_ = 0.999 **p**_**2**_ **= 0.013** **p**_**3**_ **= 0.023**
SCP Δf	0.2584 ± 0.1120	0.2620 ± 0.1042	0.2647 ± 0.1206	0.940^b^
SCP Δα	0.4147 ± 0.2310	0.3750 (0.0792–1.1809)	0.3662 (0.0897–0.7303)	0.701^a^
SCP *A*	0.2329 (0.0991–1.0572)	0.2670 (0.0847–1.0096	0.2291 (0.0769–0.9199)	0.698^a^
DCP D_0_	1.9173 (1.8821–1.9299)	1.9118 (1.8398–1.9258)	1.9216 (1.8481–1.9333)	**0.001** ^**a**^ p_1_ = 0.240 **p**_**2**_ **= 0.001** p_3_ = 0.053
DCP D_1_	1.8812 ± 0.0105	1.8795 (1.8342–1.8962)	1.8913 (1.8605–1.9055)	**0.001** ^**a**^ p_1_ = 0.747 **p**_**2**_ **= 0.001** **p**_**3**_ **= 0.001**
DCP D_2_	1.8595 (1.8215–1.8724)	1.8552 ± 0.0119	1.8697 ± 0.0098	**0.001** ^**a**^ p_1_ = 0.061 **p**_**2**_ **= 0.002** p_3_ = 0.129
DCP D_−5_	2.9206 (2.1461–4.0523)	3.0014 ± 0.5329	3.0102 ± 0.5822	0.999^a^
DCP D_5_	1.8206 ± 0.0117	1.8198 ± 0.0157	1.8406 ± 0.0154	**0.001** ^**b**^ p_1_ = 0.958 **p**_**2**_ **= 0.001** **p**_**3**_ **= 0.001**
DCP Δf	0.3202 (0.0858–0.3928)	0.2872 ± 0.0869	0.3211 (0.1265–0.5226)	0.526^a^
DCP Δα	0.4693 ± 0.1771	0.4432 ± 0.1733	0.4250 ± 0.1919	0.305^b^
DCP *A*	0.1605 (0.0693–0.7140)	0.1618 (0.0829–1.0691)	0.1331 (0.0680–0.6716)	0.139^a^
Lacunarity parameter b				
SCP	1.704 ± 0.175	1.712 ± 0.177	1.733 ± 0.187	0.618^b^
DCP	1.576 ± 0.189	1.555 ± 0.204	1.603 (0.958–2.143)	0.874^a^

*MWO* migraine without aura, *MWA* migraine with aura, *SCP* superficial capillary plexus, *DCP* deep capillary plexus, a: Kruskal-Wallis test, b: One Way ANOVA test, p_1_: the difference between MWO and MWA group, p_2_: the difference between MWA and control group, p_3_: the difference between MWO and control group, p_1,_p_2,_ and p_3_ represent the adjusted p-values.

### Lacunarity analysis

**Fig. 5:**
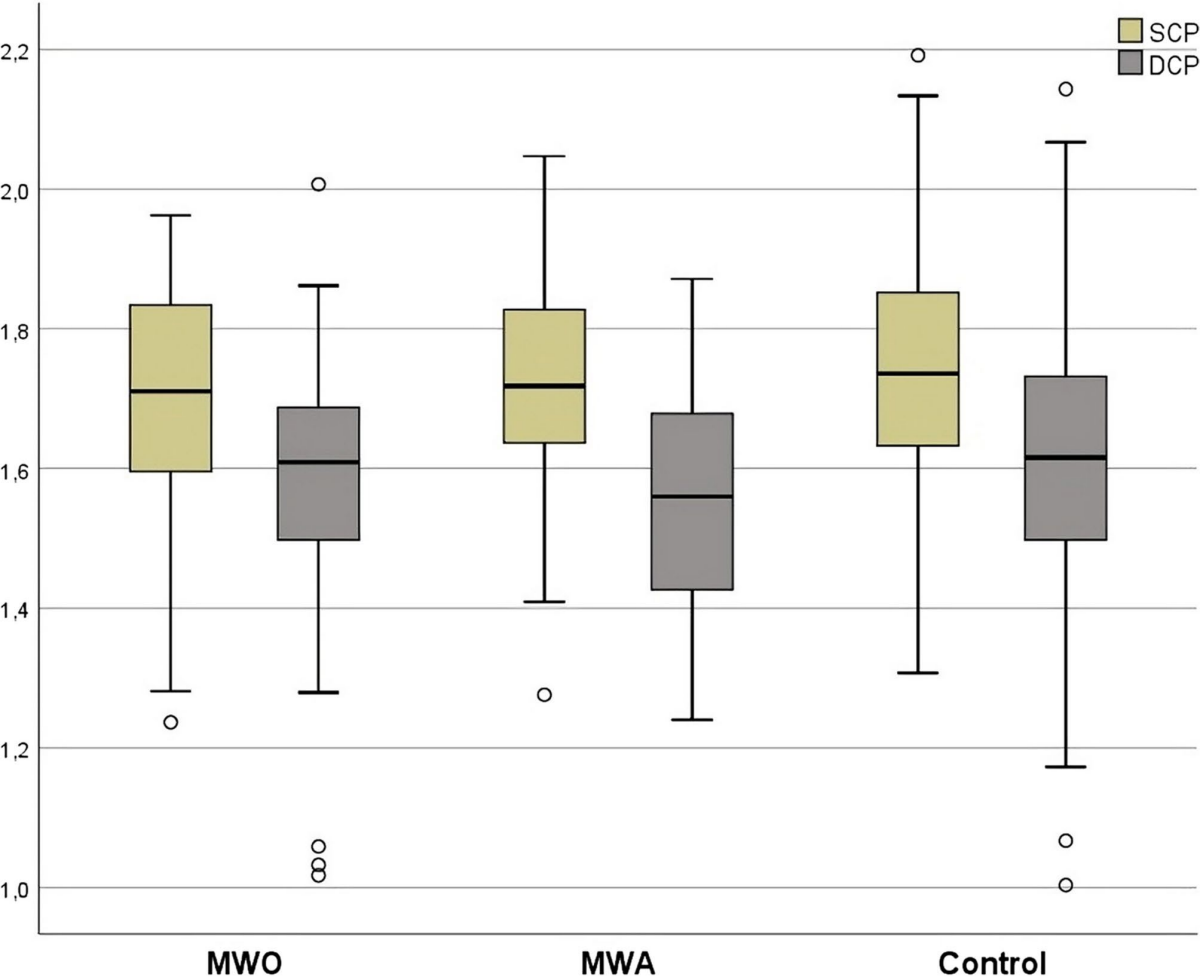
Distribution of lacunarity values (parameter b) for SCP and DCP in migraine and control groups

Lacunarity values (parameter b) did not show statistically significant differences between the groups in either SCP or DCP. Despite a trend toward lower b values in the MWA group, indicating potentially higher lacunarity, this difference did not reach statistical significance (Table 5). Figure 5 shows box plots of the lacunarity parameter for SCP and DCP.

## Discussion

In this study, we demonstrated that patients with migraine exhibit altered multifractal and lacunarity characteristics of the retinal microvasculature compared with healthy controls. These findings suggest that structural complexity and heterogeneity of the retinal vascular network are reduced in migraine, supporting the hypothesis of microvascular involvement in migraine pathophysiology. Given the close similarities in vascular regulation between the retina and the brain—such as the presence of blood-retina and blood-brain barriers and overlapping neurotransmitter profiles—the retinal microvasculature provides a unique and accessible window into cerebral vascular function [23, 24]. OCTA, a non-invasive, high-resolution imaging modality, may provide clinically relevant information by detecting subtle perfusion changes and microvascular alterations in patients with migraine.

Previous studies investigating retinal microvascular changes in migraine have yielded heterogeneous findings, partly due to differences in the timing of OCTA imaging. Ictal-phase analyses demonstrated transient vascular alterations. Podraza et al. compared ictal and interictal scans within the same patients and found a 7% reduction in parafoveal Vessel Flux Index during migraine attacks, while interictal scans revealed enduring differences in foveal perfusion (0.093 ± 0.023 vs. 0.107 ± 0.021; *p* = 0.003) and FAZ circularity (0.686 ± 0.088 vs. 0.629 ± 0.120; *p* = 0.004) between migraine with aura and without aura [24]. Similarly, Romozzi et al. (2025) observed that foveal choriocapillaris vessel density significantly decreased during ictal phases compared with interictal phases (63.3 ± 2.47% vs. 64.9 ± 2.79%, *p* = 0.0019), along with FAZ enlargement and reductions in peripapillary vessel densities [14]. 

In contrast, interictal studies—including the present work—have consistently identified more stable microvascular alterations. Our findings of reduced fractal dimension and lacunarity in migraine patients align with the systematic review and meta-analysis by Pang et al. (2023), which reported significantly reduced superficial (SMD = − 0.30, *p* = 0.0001) and deep (SMD = − 0.61, *p* = 0.02) macular vascular densities, along with enlarged FAZ (SMD = 0.56, *p* < 0.0001) [13]. These results likely reflect chronic microvascular remodeling rather than acute hemodynamic fluctuations. This distinction underscores the necessity of clearly defining OCTA acquisition timing relative to migraine attacks, as ictal and interictal findings represent distinct but complementary aspects of migraine pathophysiology.

Our results demonstrate that migraine patients, particularly those with aura, show significant alterations in both the superficial and deep capillary plexuses. MWA patients exhibited reduced VD, VAD, VLD, and BD, indicating impaired microvascular perfusion and network complexity. These findings are consistent with the study by He et al., which demonstrated reduced central and parafoveal vascular density in both SCP and DCP among migraine patients [23]. Similarly, Podraza et al. reported decreased vessel density and perfusion parameters in the macular region of the migraine with aura group, suggesting microvascular compromise potentially related to ischemic or vasospastic mechanisms [24]. 

Alterations in FAZ morphology were prominent in migraine patients, particularly those with aura. The FAZ area and perimeter were significantly enlarged in MWA patients, and FD-300—a measure of capillary density in the peri-FAZ region—was reduced, especially in the deep capillary plexus. These findings align with earlier studies suggesting that repeated hypoperfusion or vasospasm episodes may induce capillary dropout and morphological disruption of the FAZ in migraine patients [13, 25]. The increased FAZ acircularity in MWA also points to microvascular remodeling and structural instability. However, similar trends in MWO patients suggest that FAZ irregularity may not be specific to aura subtypes.

FD analysis provided more profound insight into the retinal vascular network’s complexity. The reduced D0, D1, and D2 values observed in MWA patients reflect a significant loss in structural complexity and self-similarity across scales. This vulnerability of the DCP may be attributed to its role as a “watershed zone” with limited oxygen supply and the high metabolic demand imposed by the outer retina, making it more susceptible to hypoxic or ischemic stress. Comparable findings have been reported in other ischemic conditions: Zhu et al. demonstrated that reduced fractal dimension in diabetic retinopathy reflects early capillary dropout and microvascular degradation [26]. 

Multifractal spectrum (D(q)) analysis further supported these observations. The MWA group exhibited the lowest D(q) values, particularly in the negative q domain, suggesting reduced contributions from low-density vascular regions. Although multifractal asymmetry (A), Δf, and Δα did not reach statistical significance, the consistent downward shift in the D(q) curve across the MWA group indicates a generalized loss of vascular complexity. In line with our results, Zhu et al. reported a leftward shift of the multifractal spectrum at the DCP level in diabetic retinopathy, while Gould et al. emphasized that multifractal analysis provides greater sensitivity than monofractal metrics in detecting subtle and spatially heterogeneous vascular alterations [22, 26]. 

Lacunarity analysis provided complementary insights into spatial organization. While group differences were not statistically significant, a trend toward increased lacunarity (lower *b* values) was observed in the MWA group, indicating greater heterogeneity and irregularity in capillary spacing. This pattern may indicate localized dropout or discontinuities within the vascular meshwork, reflecting subclinical remodeling processes. Popović et al. reported similarly elevated lacunarity in proliferative diabetic retinopathy, while Tălu et al. noted altered lacunarity in amblyopia, supporting its potential as a biomarker of microvascular disruption and structural instability [27, 28]. 

When considered collectively, our findings—particularly the reduction in vascular complexity and the trend toward increased spatial heterogeneity in MWA patients—underscore the utility of advanced morphometric OCTA metrics such as fractal dimension, multifractal spectrum, and lacunarity in detecting subtle microvascular alterations. These changes were most pronounced in the deep capillary plexus, a region known for its vulnerability to ischemic stress due to its limited oxygen supply and high metabolic demand.

Although not all metrics reached statistical significance, the consistent downward shift in multifractal spectra and the tendency toward increased lacunarity in MWA support the hypothesis of subclinical ischemic damage, particularly in patients with aura. These converging trends across multiple descriptors point to chronic or recurrent microvascular dysfunction that may underlie aura-related pathophysiological processes.

Our study highlights the potential of OCTA-based quantitative morphometric analysis as a non-invasive approach to evaluate retinal microvascular alterations in migraine. Future longitudinal studies are warranted to determine whether these retinal biomarkers can predict disease progression or monitor therapeutic response.

This study has several limitations. First, its cross-sectional design precludes causal inferences. Although sufficient to detect group differences, the overall sample size—particularly in the migraine with aura subgroup —may have limited the statistical power of multifractal and lacunarity analyses. Another important limitation is the heterogeneity of the migraine cohort. While attack frequency was documented, patients were not stratified into low-frequency episodic, high-frequency episodic, or chronic migraine subgroups. This variability in disease severity may have influenced retinal vascular changes, and future studies should assess the role of disease frequency and chronicity in shaping microvascular remodeling.

Methodological considerations should also be acknowledged. Although validated, manual image processing and segmentation may have introduced variability. Preventive migraine medications (e.g., beta-blockers, calcium channel blockers, antiepileptics) were applied as exclusion criteria, minimizing potential confounding. However, the influence of acute medications such as triptans could not be fully assessed, and their vascular effects remain an important consideration for future research.

In conclusion, OCTA-based fractal and lacunarity analyses revealed significant differences in retinal microvascular structure, particularly in patients with migraine with aura. Fractal dimension metrics demonstrated reduced vascular complexity, while lacunarity trends suggested increased spatial heterogeneity, together pointing to underlying neurovascular dysregulation and subclinical ischemia. By combining multifractal and lacunarity approaches, our study provides novel morphometric evidence of microvascular alterations in migraine, with changes most pronounced in the deep capillary plexus. These findings highlight the potential of quantitative OCTA imaging as a non-invasive biomarker for migraine research. Future longitudinal studies are warranted to determine whether these vascular alterations can predict disease progression, differentiate subtypes, or monitor therapeutic response.

## Data Availability

The datasets generated and analyzed during the current study are not publicly available but are available from the corresponding author on reasonable request.
