# Correction: Multifractal and lacunarity features of retinal microvasculature in migraine: an optical coherence tomography angiography study

**DOI:** 10.1186/s12886-025-04508-8

**Published:** 2025-11-11

**Authors:** Abdülcemal Gürpınar, Yüksel Süllü

**Affiliations:** 1 Department of Ophthalmology, Ordu State Hospital, Ordu, 55200 Turkey; 2 Department of Ophthalmology, Ondokuz Mayıs University, Samsun, 55139 Turkey

**Correction: BMC Ophthalmol 25**,** 274 (2025)**

10.1186/s12886-025-04407-y

In the abstract of this article, the sentence currently reads:

“A total of 177 eyes from 56 migraine patients (35 without aura, 21 with aura) and 102 eyes from 51 healthy controls were included in the study.”

This should be corrected to:

“A total of 112 eyes from 56 migraine patients (35 MWO, 21 MWA) and 102 eyes from 51 healthy controls were included in the study.”

This was a typographical error and does not affect the results, analyses, or conclusions of the study.

The original article has been updated.

The authors would like to apologize for any inconvenience caused.

